# Efficacy and safety of saxagliptin in patients with type 2 diabetes: A systematic review and meta-analysis

**DOI:** 10.1371/journal.pone.0197321

**Published:** 2018-05-22

**Authors:** Peng Men, Xiao-tong Li, Hui-lin Tang, Suo-di Zhai

**Affiliations:** Department of Pharmacy, Peking University Third Hospital, Beijing, China; Weill Cornell Medical College Qatar, QATAR

## Abstract

**Objective:**

To evaluate the comparative efficacy and safety of saxagliptin for type 2 diabetes (T2D).

**Methods:**

A systematic search of PubMed, Embase, the Cochrane Library, Web of Science, ClinicalTrials.gov and two Chinese databases for randomized controlled trials (RCTs) comparing saxagliptin with placebo or active comparators was performed up to July 2017. A complementary search was done to cover literature until March 2018. For continuous data, estimates were pooled using inverse variance methodology to calculate weighted mean differences (WMDs). Dichotomous data were presented as Mantel-Haenzel risk ratios (RRs).

**Results:**

Thirty-nine references of 30 RCTs involving 29,938 patients were analyzed. Compared with placebo, saxagliptin significantly reduced glycated hemoglobin (HbA1c, WMD -0.52%, 95% CI -0.60 to -0.44) and fasting plasma glucose (WMD -13.78 mg/dL, 95% CI -15.31 to -12.25), and increased the proportion of patients achieving HbA1c <7% (RR 1.64, 95% CI 1.53 to 1.75). When combined with submaximal-dose metformin, saxagliptin significantly increased the proportion of patients achieving HbA1c <7% compared with acarbose (RR 2.38, 95% CI 1.17 to 4.83) and uptitrated metformin (RR 1.30, 95% CI 1.04 to 1.63). Saxagliptin was similar to other DPP-4 inhibitors but inferior to liraglutide and dapagliflozin on glycemic control. Saxagliptin significantly decreased the incidences of overall adverse events compared with acarbose (RR 0.71, 95% CI 0.57 to 0.89) and liraglutide (RR 0.41, 95% CI 0.24 to 0.71) when added to metformin. Weight gain and hypoglycemia with saxagliptin was slightly but significantly higher than placebo and lower than sulfonylureas. Saxagliptin did not increase the risk of arthralgia, heart failure, pancreatitis and other adverse events.

**Conclusions:**

Generally, saxagliptin has similar efficacy compared with most oral antidiabetic drugs and may be more effective than acarbose, while having a better safety profile than both acarbose and sulfonylureas.

## Introduction

Type 2 diabetes (T2D) is a chronic disease rapidly increasing in prevalence that imposing enormous medical and economic burdens on on individuals, families, and national health systems worldwide. It is estimated that approximately 415 million people in the world had diabetes in 2015, and this figure is projected to increase to 642 million by 2040 [1]. Health spending on diabetes accounted for 11.6% of total health expenditure worldwide in 2015 [2]. A diversity of antidiabetic drugs to treat the condition is now available, including metformin, sulfonylureas, thiazolidinediones, α-glucosidase inhibitors, prandial glucose regulators, sodium-glucose cotransporter 2 (SGLT2) inhibitors, and insulin in various forms. Because of the progressive nature of diabetes, clinicians and patients often experience difficulty in achieving and sustaining glycemic control. Utilization of antidiabetic drugs should be based on the individual patient’s characteristics and preferences and balance the need to optimize the benifits of glycaemic control with the need to limit the risk of adverse effects.

Dipeptidyl peptidase 4 (DPP-4) inhibitors (gliptins) are a relatively new class of oral antidiabetic drugs for the treatment of type 2 diabetes. They act by increasing postprandial concentrations of glucagon-like peptide-1 (GLP-1) and glucose-dependent insulinotropic peptide (GIP) [3–4]. GLP-1 and GIP stimulate insulin secretion in a glucose-dependent manner, suppressing glucagon secretion and slowing gastric emptying. The American Diabetes Association and the European Association for the Study of Diabetes have advocated the use of DPP-4 inhibitors as first-line agent in circumstances where metformin is contraindicated or not tolerated, or within a dual or triple agent regimen [5]. Saxagliptin is an orally active, once-daily, selective and reversible inhibitor of DPP-4 enzyme indicated/approved as an adjunct to diet and exercise to improve glycemic control in adults with type 2 diabetes [6,7].

With the growing number of pharmacological options for treating type 2 diabetes, there is a need for state-of-the-art evidence to inform clinical decisions about differences among the various medications. Recent systematic reviews and meta analyses of DPP-4 inhibitors have mainly focused on the clinical profiles of these drugs as a whole class [8–11]. For saxagliptin, only one meta-analysis [12] examined its efficacy based on 14 phase 2 and 3 trials, without systematically database searches. This systematic review synthesized currently available evidence to provide a better understanding of the comparative efficacy and safety of saxagliptin in treating type 2 diabetes.

## Methods

### Data sources and search strategy

This meta-analysis of the available data on saxagliptin was undertaken using a predetermined protocol, and is reported in accordance with the Preferred Reporting Items for Systematic Reviews and Meta-Analyses (PRISMA) statement (see S1 Text) [13]. Relevant studies for the analysis were selected by searching PubMed, Embase, the Cochrane Library, Web of Science, and 2 Chinese databases [China National Knowledge Infrastructure (CNKI) and Chinese Biomedical Literature Database (CBM)] up to the end of July 2017. A complementary search was performed in order to include the most recent articles (published before March 2018). The generic drug name “saxagliptin” was used as the only search term. If necessary, specific filters for retrieving randomized controlled trials (RCTs) conducted in humans were incorporated into the search string. We considered all potentially eligible studies for review, irrespective of the primary outcomes or language. We also performed a manual search using the reference lists of published reviews of saxagliptin.

The research questions and eligibility criteria for the systematic review conformed with the PICOS (participants, interventions, comparators, outcomes and study design) approach. Studies meeting the following criteria were considered for inclusion:

- *Participants*: patients over 18 years of age with type 2 diabetes.
- *Interventions*: saxagliptin used in the treatment of type 2 diabetes (as monotherapy or in dual or triple therapy).
- *Comparators*: placebo or other active antidiabetic interventions (as monotherapy or in dual or triple therapy).
- *Outcomes*: glycated hemoglobin (HbA1c), proportion of patients achieving HbA1c targets of <7%, fasting plasma glucose (FPG) concentration, overall and serious adverse events, body weight, confirmed hypoglycemia, heart failure, pancreatitis, arthralgia, and other adverse events [hypertension, urinary tract infection, upper respiratory tract infection, nasopharyngitis]. For continuous data, the mean change from baseline was examined.
- *Study type*: RCTs involving >150 patients.

### Study selection and data extraction

The titles and abstracts of all retrieved citations were independently screened by 2 reviewers to identify potentially relevant studies. The full texts of relevant citations were then retrieved to determine their suitability for inclusion. If there were any discrepancies between the 2 reviewers, a third reviewer became involved.

Data were independently abstracted by the 2 principal reviewers and any discrepancies were resolved by consensus. The data extracted included study characteristics (design, total number of participants, trial duration, antidiabetic treatments, patient inclusion and exclusion criteria), patient demographics (age, sex, baseline HbA1c levels), and pre-specified efficacy and safety outcomes. For extension studies, if the treatment assignment was switched from placebo to saxagliptin, only the outcome data up to that point were documented. Only information that related to dosages currently approved by the US Food and Drug Administration (FDA) or the European Medicines Agency (EMA) were abstracted. Corresponding authors were contacted for data not provided within studies, or when outcomes were presented in an unsuitable format for data synthesis.

### Quality assessment

Two reviewers independently applied the Cochrane Risk of Bias tool to assessed the risk of bias in the RCTs. The following methodological features relevant to the minimization of bias were assessed: randomization, random allocation concealment, masking of treatment allocation, blinding, incomplete outcomes data, selective reporting, and other items. The following judgements were used: low risk, high risk, or unclear risk (either lack of information or uncertainty regarding the potential for bias). Disagreements were resolved by consensus with a third reviewer.

### Data synthesis and analysis

Outcomes were pooled using Review Manager 5.2 software (RevMan, Cochrane, London, UK). For continuous data, estimates were pooled using inverse variance methodology to calculate weighted mean differences (WMDs). Dichotomous data were presented as Mantel-Haenzel risk ratios (RRs). All results were estimated from each study with 95% confidence intervals (CIs). Heterogeneity was assessed using the chi-square test and the I^2^ statistic. If I^2^ was 50%, a fixed-effect model with the Mantel-Haenszel method was used; otherwise, the random-effect model was adopted.

Where studies did not report standard deviations (SDs) explicitly, these were derived from the available published information. If possible, standard errors (SEs) were calculated from CIs. If these data were unavailable, they were derived from *p*-values for changes from baseline. All trials were included in at least one of the analyses, and each trial could be used in multiple sets of analyses. Data were reported first from studies involving placebo comparisons, and then from studies involving active comparators.

## Results

**Fig 1:**
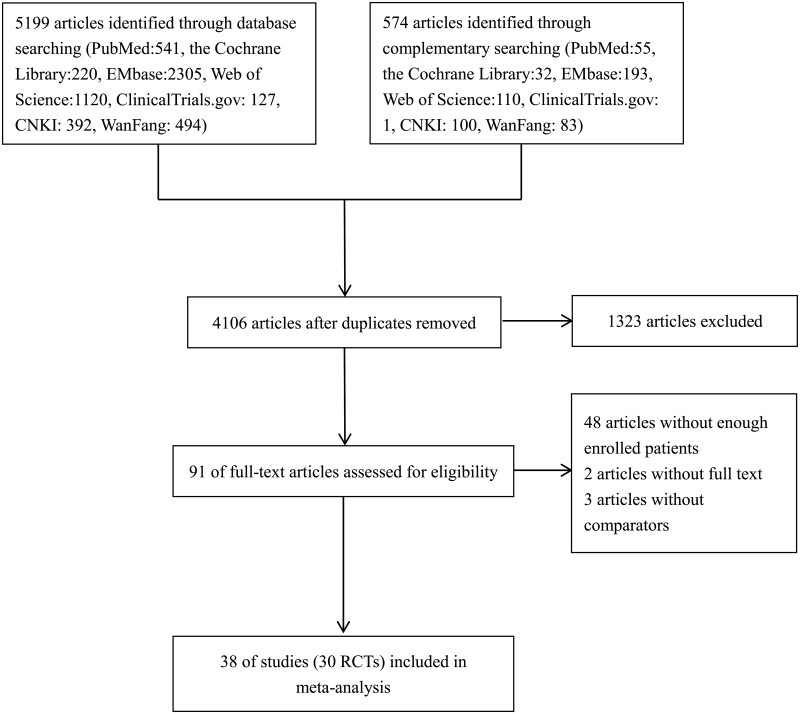
Selection process for articles in the systematic review.

The selection process for articles included in the systematic review is shown in Fig 1. From the 5773 citations identified by the initial and complementary literature searching, 38 references [14–51], including 30 trials involving 29,938 participants, ultimately met the inclusion criteria for the meta-analysis. The study characteristics and the demographics of the patient populations in the retrieved studies were comparable (Table 1). Enrolled patients in these RCTs had a mean age of 42.0 to 72.6 years. The mean duration of type 2 diabetes ranged from 0.4 to 16.7 years, with mean baseline HbA1c levels between 7.6% and 10.7%.

**Table 1:** Patient demographics and study characteristics of the included studies.

Trial No.	Reference, year	Clinicaltrial.gov No.	Trial duration(weeks)	n	Mean age(years)	Sex (male/female)	Baseline HbA1c (%)	Baseline FPG (mg/dL)	Baseline body weight (kg)	T2D duration(years)	Interventions	Added-on to
1	Barnett et al., 2012 [14]	NCT00757588	24	455	57.3	188/267	8.7	173.4	87.2	11.9	SAXA 5 mg/d PLB	INS
	Barnett et al., 2013 [15]		52									
2	Chacra et al., 2009 [16]	NCT00313313	24	768	55.0	346/422	8.4	173.4	75.7	6.9	SAXA 2.5 mg/d SAXA 5 mg/d PLB	GLY
	Chacra et al., 2011 [17]		76									
3	Chen et al., 2017 [18]	NCT02104804	24	462	59.1	209/253	8.5	167.6	NA	13.4	SAXA 5 mg/d PLB	INS±MET
4	DeFronzo et al., 2009 [19]	NCT00121667	24	743	54.6	NA	8.0	176.0	NA	6.5	SAXA 2.5 mg/d SAXA 5 mg/d SAXA 10 mg/d PLB	MET
	Rosenstock et al., 2013 [20]		206									
5	Dou et al., 2017 [21]	NCT02273050	24	630	50.1	419/211	9.4	182.7	73.8	0.81	SAXA 5 mg/d PLB	MET
6	Du et al., 2017 [22]	NCT02243176	24	481	55.6	285/196	8.2	160.3	72.9	5.2	SAXA 5 mg/dACBO 300 mg/d	MET
7	Fonseca et al., 2012 [23]	NCT00960076	18	282	55.3	130/152	8.4	162.9	NA	6.2	SAXA 5 mg/d MET (uptitrated to 2000 mg/d)	MET
8	Frederich et al., 2012 [24]	NCT00316082	76	365	54.8	168/197	7.9	162.1	84.9	1.7	SAXA 2.5 mg/d SAXA 5 mg/d PLB	‒
9	Goke et al., 2010 [25]	NCT00575588	52	858	57.6	444/414	7.7	162.0	88.7	5.5	SAXA 5 mg/d GLPZ 5–20 mg/d	MET
	Goke et al., 2013 [26]		104									
10	Hermans et al., 2012 [27]	NCT01006590	24	286	58.7	164/122	7.8	169.8	NA	6.5	SAXA 5 mg/d MET (uptitrated to 2000 mg/d)	MET
11	Hollander et al., 2009 [28]	NCT00295633	24	565	54.0	280/285	8.3	162.9	81.1	5.2	SAXA 2.5 mg/d SAXA 5 mg/d PLB	TZD
	Hollander et al. 2011 [29]		76									
12	Jadzinsky et al., 2009 [30]	NCT00327015	24	1306	52.0	643/663	9.5	201.3	82.5	1.7	SAXA 5 mg/d SAXA 10 mg/d PLB	MET
	Pfutzner et al., 2011 [31]		76									
13	Kadowaki et al., 2017 [32]	/	16	240	63.4	139/89	8.3	163.1	65.5	15.8	SAXA 5 mg/d PLB	INS
14	Kumar et al., 2014 [33]	NCT00918879	24	213	49.1	120/93	8.3	151.2	69.6	0.9	SAXA 5 mg/d PLB	‒
15	Li et al., 2014A [34]	/	24	207	46.6	109/81	8.7	153.1	74.4	NA	SAXA 5 mg/d SITA 100mg/d VIDA 100mg/d	MET+ another OAD
16	Li et al., 2014B [35]	/	24	178	47.1	109/69	8.5	160.9	75.9	5.6	SAXA 5 mg/d VIDA 100mg/d LRAG 1.2mg/d	OAD
17	Lv et al., 2013 [36]	/	12	180	44.0	NA	7.8	150.2	82.0	0.5	SAXA 5 mg/d ACBO 150 mg/d	MET
18	Matthaei et al., 2015 [37]	NCT01619059	24	315	54.6	149/166	7.9	161.0	NA	7.7	SAXA 5mg/d PLB	DAPA+MET
	Matthaei et al., 2016 [38]		52									
19	Moses et al., 2014 [39]	NCT01128153	24	257	57.0	154/103	8.3	159.3	81.4	-	SAXA 5 mg/d PLB	MET+SU
20	Nowicki et al., 2011 [40,41]	NCT00614939	12, 52	170	66.5	73/97	8.3	178.3	82.9	16.7	SAXA 2.5 mg/d PLB	‒
21	Pan et al., 2012 [42]	NCT00698932	24	568	51.4	315/253	8.2	164.7	69.2	1.0	SAXA 5 mg/d PLB	‒
22	Rosenstock et al., 2008 [43]	NCT00950599	12	338	54.0	197/141	7.9	164.7	89.9	1.1	SAXA 2.5 mg/d SAXA 5 mg/d SAXA 10 mg/d SAXA 20 mg/d SAXA 40 mg/d PLB	‒
			6	85	52.1	50/35	7.7	148.7	91.2	0.4	SAXA 100 mg/d PLB	‒
23	Rosenstock et al., 2009 [44]	NCT00121641	24	401	53.5	204/197	7.9	175.0	89.8	2.6	SAXA 2.5 mg/d SAXA 5 mg/d SAXA 10 mg/d PLB	‒
	Rosenstock et al., 2013 [20]			68	49.1	79/82	10.7	241.0	91.4	3.1	SAXA 10 mg/d	‒
24	Rosenstock et al., 2015 [45]	NCT01606007	24	534	54.0	268/266	8.9	186	NA	7.6	SAXA 5 mg/d DAPA 10 mg/d SAXA 5 mg/d + DAPA 10 mg/d	MET
25	Scheen et al., 2010 [46]	NCT00666458	18	801	58.4	392/409	7.7	161.1	NA	6.3	SAXA 5 mg/d SITA 100mg/d	MET
26	Schernthaner et al., 2015 [47]	NCT01006603	52	720	72.6	445/275	7.6	NA	NA	7.6	SAXA 5 mg/d ≤6 mg/d	MET
27	Scirica et al., 2013 [48]	NCT01107886	109	16492	65.1	11037/5455	8.0	156.5	87.9	10.3	SAXA 5 mg/d PLB	‒
28	White et al., 2014 [49]	NCT00885378	12	160	55.7	85/75	8.0	163.1	NA	6.0	SAXA 5 mg/d PLB	MET
29	Xia et al., 2014 [50]	/	12	240	42.0	126/114	8.3	157.5	NA	1.0	SAXA 5 mg/d REGL 3 mg/d	MET
30	Yang et al., 2011 [51]	NCT00661362	24	570	54.1	275/295	7.9	159.3	69.0	5.1	SAXA 5 mg/d PLB	MET

ACBO, acarbose; DAPA, dapagliflozin; GLMR, glimepiride; GLPZ, glipizide; GLY, glyburide; INS, insulin; LINA, linagliptin; MET, metformin; NA, not available; PLB, placebo; REGL, repaglinide; SAXA, saxagliptin; SITA, sitagliptin; SU, sulfonylurea; TZD, thiazolidinedione; VIDA, vildagliptin.

### Quality of the included studies

A risk of bias summary is shown in S1a Fig, and an assessment of the risk of bias for each of the studies selected is shown in S1b Fig. Random sequence generation was adequate in 25 trials, and allocation concealment was adequately described in 16 trials. Two trials were considered to be at high risk of performance and detection bias. All studies were judged to be at low risk of attrition, reporting and other bias.

### Glycemic control

#### Glycated hemoglobin (HbA1c)

**Fig 2:**
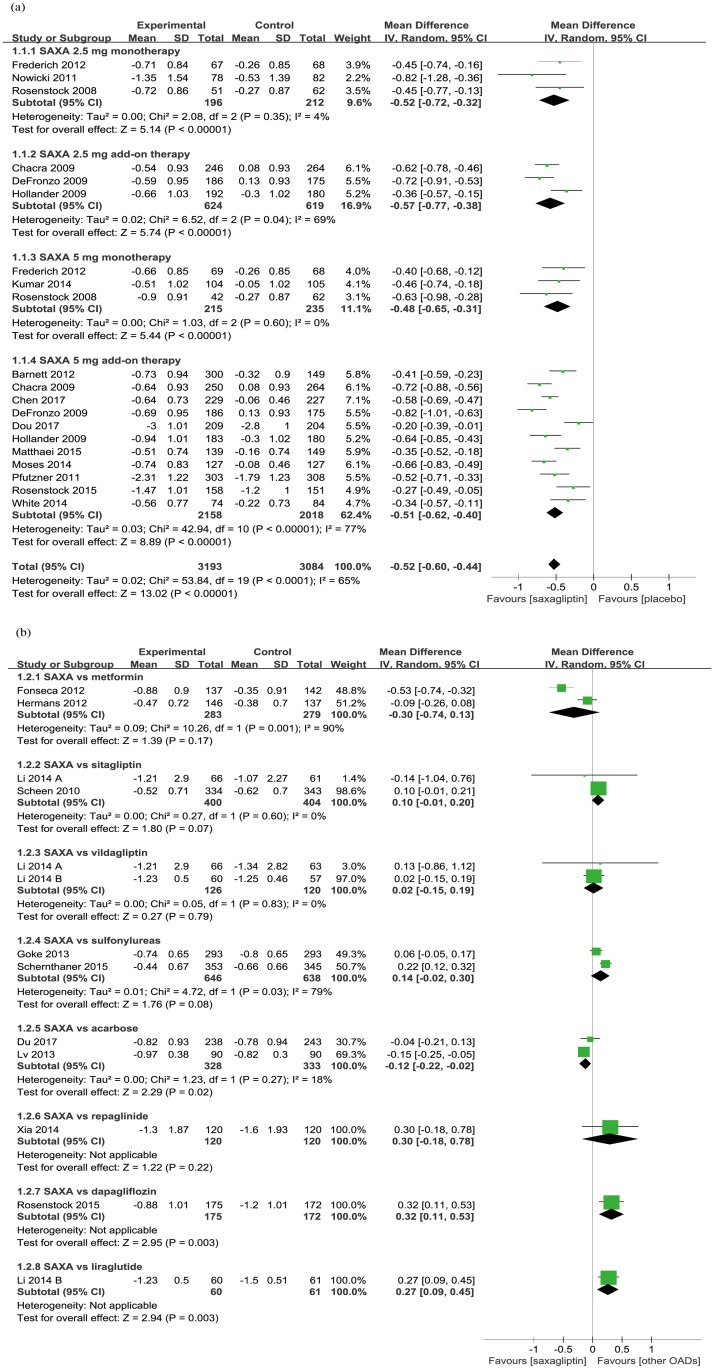
Mean change of HbA1c from baseline (a. saxagliptin vs placebo; b. saxagliptin vs active comparators).

In comparison with placebo, saxagliptin produced significantly greater reductions in HbA1c (WMD −0.52%, 95% CI −0.60 to −0.44; *p* < 0.00001; Fig 2a), whether used as monotherapy (WMD −0.50%, 95% CI −0.62 to −0.37; *p* < 0.00001) or add-on therapy (WMD −0.52%, 95% CI −0.62 to −0.43; *p* < 0.00001). Both the 2.5 mg/day and 5 mg/day dosages of saxagliptin produced significant improvements in HbA1c, with similar absolute effect sizes.

Overall, saxagliptin produced similar reduction in HbA1c compared with active comparators when added to metformin (WMD 0.01%, 95% CI −0.11 to 0.13; *p* = 0.89; Fig 2b). Saxagliptin significantly reduced HbA1c in comparison with acarbose (WMD −0.12%, 95% CI −0.22 to −0.02; *p* = 0.02), but not compared with liraglutide (WMD 0.27%, 95% CI 0.09 to 0.45; *p* = 0.003) or dapagliflozin (WMD 0.32%, 95% CI 0.11 to 0.53; *p* = 0.003). Saxagliptin produced similar reduction compared with metformin (WMD -0.30%, 95% CI -0.74 to 0.13; *p* = 0.17), sulfonylureas (WMD 0.14%, 95% CI -0.02 to 0.30; *p* = 0.08), sitagliptin (WMD 0.10%, 95% CI -0.01 to 0.20; *p* = 0.07), vildagliptin (WMD 0.02%, 95% CI -0.15 to 0.19; *p* = 0.79) and repaglinide (WMD 0.30%, 95% CI -0.18 to 0.78; *p* = 0.22).

A significantly greater proportion of patients treated with saxagliptin also achieved a target HbA1c of <7% compared with placebo (RR 1.64, 95% CI 1.53 to 1.75; *p* < 0.00001; S2a Fig), whether used as monotherapy (RR 1.53, 95% CI 1.32 to 1.77; p < 0.00001) or add-on therapy (RR 1.67, 95% CI 1.55 to 1.81; *p* < 0.00001). Similar results was shown when compared with metformin (RR 1.30, 95% CI 1.04 to 1.63; *p* = 0.02) and acarbose (RR 2.38, 95% CI 1.17 to 4.83; *p* = 0.02). However, no significant differences were observed in comparisons of saxagliptin with other active comparators (S2b Fig).

#### Fasting plasma glucose (FPG)

**Fig 3:**
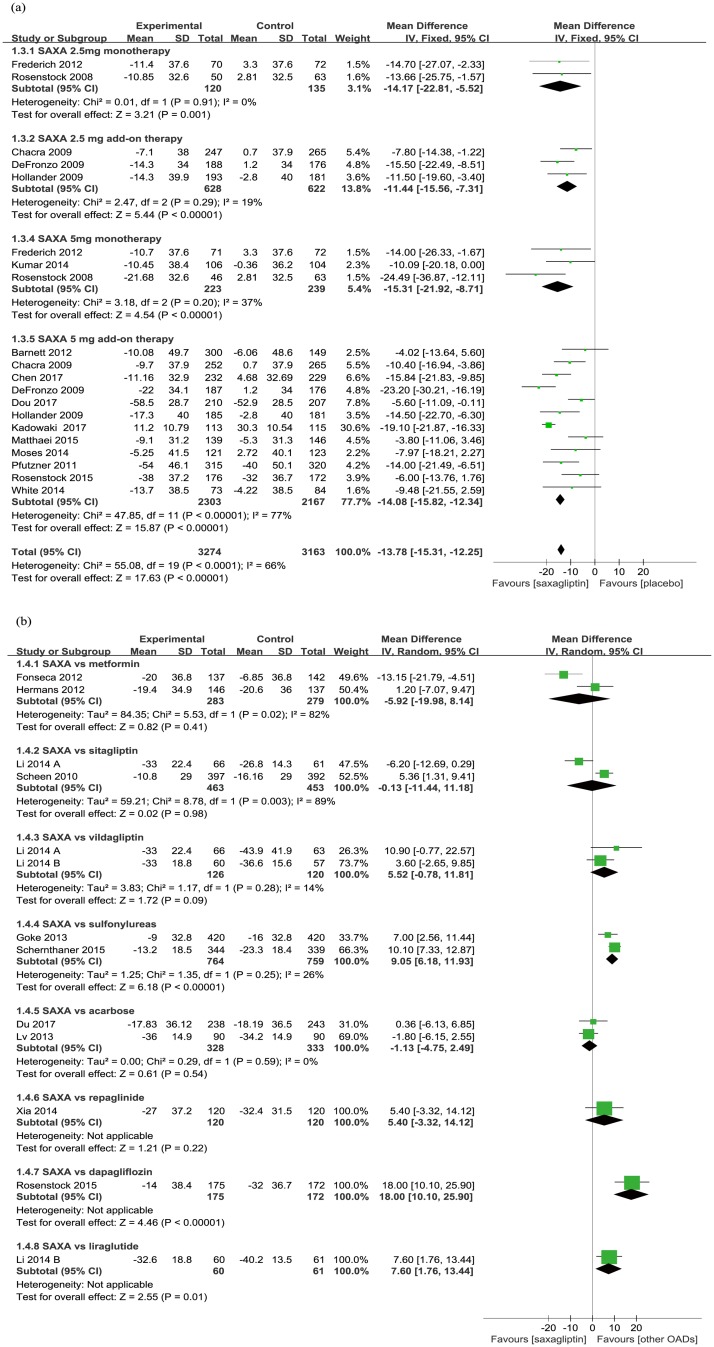
Mean change of FPG from baseline (a. saxagliptin vs placebo; b. saxagliptin vs active comparators).

In comparison with placebo, saxagliptin produced significantly greater reductions in FPG (WMD −13.78 mg/dL, 95% CI −15.31 to −12.25; *p* < 0.00001; Fig 3a), whether used as monotherapy (WMD −14.89 mg/dL, 95% CI −20.14 to −9.64; *p* < 0.00001) or add-on therapy to other antidiabetic agents (WMD −13.68 mg/dL, 95% CI −15.28 to −12.08; *p* < 0.00001). Both the 2.5 mg/day and 5 mg/day dosages of saxagliptin produced significant improvements in FPG.

When added to metformin, saxagliptin produced a significantly smaller reduction in FPG compared with sulfonylureas (WMD 9.05 mg/dL, 95% CI 6.18 to 11.93; *p* < 0.00001), liraglutide (WMD 7.60 mg/dL, 95% CI 1.76 to 13.44; *p* = 0.01) and dapagliflozin (WMD 18.00 mg/dL, 95% CI 10.10 to 25.90; *p <* 0.00001). However, no significant differences were observed when saxagliptin was compared with other active comparators (Fig 3b), including sitagliptin (WMD -0.13 mg/dL, 95% CI -11.44 to 11.18; *p =* 0.98) and vildaglitpin (WMD 5.52 mg/dL, 95% CI -0.78 to 11.81; *p =* 0.09).

### Non-glycemic outcomes

#### Overall and serious adverse events

**Fig 4:**
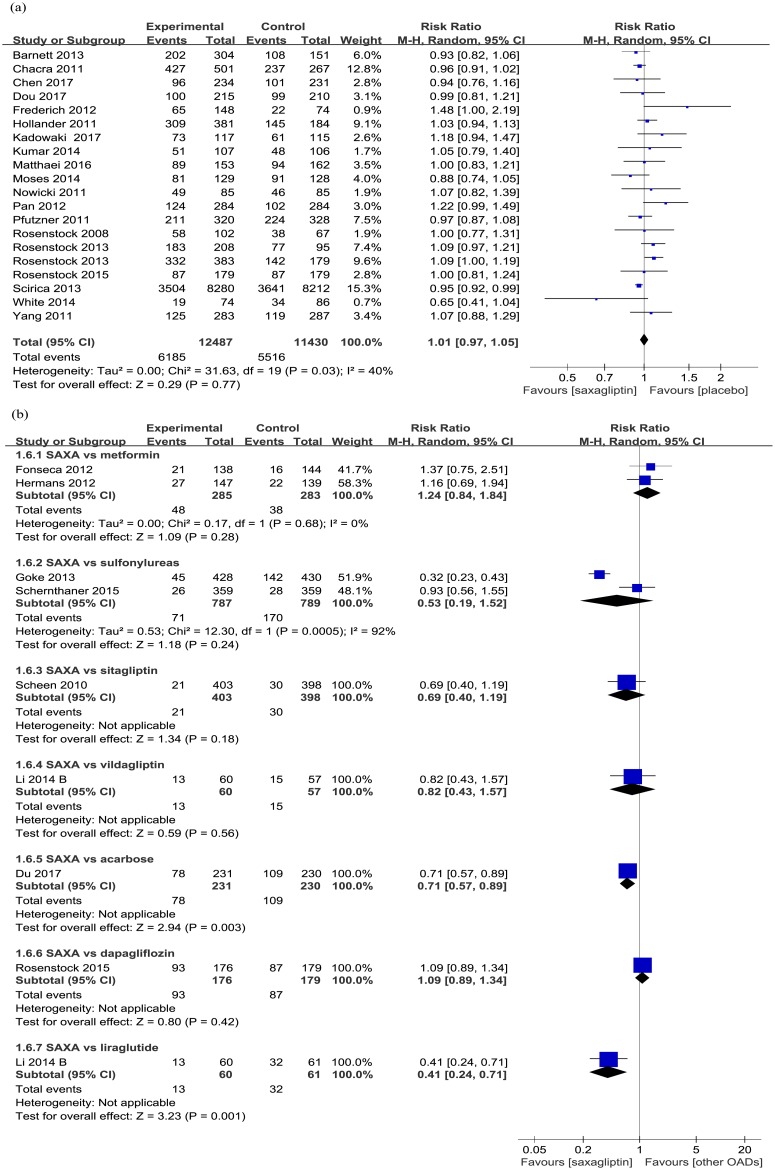
Overall adverse events (a. saxagliptin vs placebo; b. saxagliptin vs active comparators).

Generally, the incidences of overall (*versus* placobo: RR 1.01, 95% CI 0.97 to 1.05; *p* = 0.77; Fig 4a) and severe (*versus* placobo: RR 1.01, 95% CI 0.96 to 1.06; *p* = 0.78; S3 Fig) treatment-related adverse events did not increase in the treatment of saxagliptin. Moreover, saxagliptin significantly reduced overall adverse events compared with acarbose (RR 0.71, 95% CI 0.57 to 0.89; *p* = 0.03; Fig 4b) and liraglutide (RR 0.41, 95% CI 0.24 to 0.71; *p* = 0.001) when added to metformin.

#### Hypoglycemia

**Fig 5:**
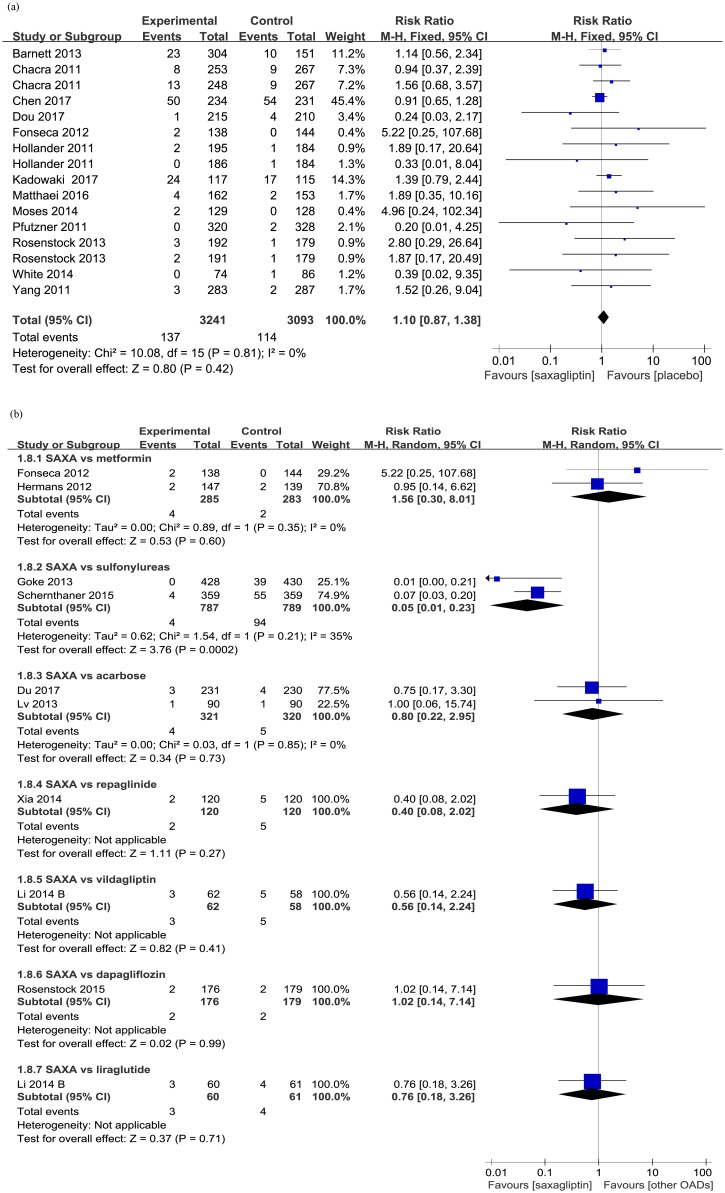
Hypoglycemia (a. saxagliptin vs placebo; b. saxagliptin vs active comparators).

Compared with placebo, saxaglitpin significantly but slightly increased the incidences of hypoglycemia (RR 1.13, 95% CI 1.05 to 1.21; *p* = 0.0009; Fig 5a). Compared with sulfonylureas, saxaglitpin significantly reduced the risk of hypoglycemia by 95% (RR 0.05, 95% CI 0.01 to 0.23; *p* = 0.0002; Fig 5b). No significant differences were observed in comparison with other active comparators, including other DPP-4 inhibitors.

#### Body weight

In comparison with placebo, treatment with saxaglipitin was associated with a significant but slight increase of body weight (WMD 0.42 kg, 95% CI 0.26 to 0.59; *p* < 0.00001; S4a Fig). Saxagliptin was inferior to liraglutide (WMD 5.10 kg, 95% CI 1.66 to 8.54; *p* = 0.004; S4b Fig) and dapagliflozin (WMD 2.40 kg, 95% CI 1.69 to 3.11; *p* < 0.00001). However, treatment with saxagliptin was associated with significantly less effect on body weight than sulfonylureas (WMD −2.34 kg, 95% CI −3.31 to −1.36; *p* < 0.00001). In comparison with other DPP-4 inhibitors, changes in body weight were similar.

#### Other adverse events

The incidences of pancreatitis (RR 1.13, 95% CI 0.65 to 1.96; *p* = 0.66; S5a Fig) and heart failure (RR 0.99, 95% CI 0.89 to 1.10; *p* = 0.85; S5b Fig) did not differ significantly between saxagliptin and controls (placebo and sulfonylureas). The risk of arthralgia did not differ significantly between saxagliptin and placebo (RR 1.02, 95% CI 0.92 to 1.13; *p* = 0.66; S5c Fig), Compared with sitagliptin, saxagliptin could significantly reduced the risk of arthralgia (RR 0.20, 95% CI 0.04 to 0.90; *p* = 0.04; S5d Fig), but not compared with other active treatments. Treatment with saxagliptin was not associated with any increased risks of upper respiratory tract infection, urinary tract infection and nasopharyngitis compared with both placebo and active comparators (*p* > 0.05). See S5e–S5j Fig. for forest plots of adverse events above.

## Discussion

This meta-analysis of the available literature on saxagliptin aimed to assess its clinical efficacy and safety in patients with type 2 diabetes. The findings of the analysis indicate that treatment with saxagliptin can lead to significant decreases of HbA1c and FPG compared with placebo, both when given as monotherapy or add-on therapy to other treatments, including metformin, sulfonylureas, thiazolidinediones, dapagliflozin and insulin. Mean placebo-adjusted HbA1c and FPG levels in saxagliptin add-on therapy were lowered by comparable amounts to saxagliptin monotherapy. When combined with submaximal-dose metformin, saxagliptin significantly increased the proportion of patients achieving HbA1c <7% compared with acarbose and uptitrated metformin. Generally, efficacy on glycemic control of saxagliptin was similar to sitaglitpin and vildagliptin, while inferior to liraglutide and dapagliflozin. More direct comparisons with other active comparators in future trials may provide further evidence of the efficacy of saxagliptin in comparison with other active treatments.

Saxagliptin is generally well tolerated, with no increased risk of overall and serious adverse events as monotherapy and combination therapy. Additionally, saxagliptin group experienced less overall adverse events than acarbose and liraglutide groups when combined with metformin, indicating its favorable safety profile in patients with T2D. Compared with sulfonylureas, saxagliptin had a significant better effect on hypoglycemia and body weight gain. Treatment of saxagliptin was shown to be with a minimal increase of hypoglycemia, which mainly depending on the result from the included Saxagliptin Assessment of Vascular Outcomes Recorded in Patients with Diabetes Mellitus -Thrombolysis in Myocardial Infarction 53 (SAVOR-TIMI 53) trial. Patients in that trial were permitted various concomitant antihyperglycaemic therapies at the doctor’s discretion. A post-hoc analysis [52] of SAVOR-TIMI 53 trial found that hypoglycemia rates (any or major) were increased with saxagliptin in patients taking sulfonylureas, not in those taking insulin. A pooled analysis also found that saxagliptin was not associated with increased reported or confirmed hypoglycemia when use of sulfonylurea was excluded [53]. Thus, lower doses of sulfonylureas might be required to reduce risk of hypoglycaemia. Saxagliptin improves the sensitivity of pancreatic islets (α- and β-cells) to glucose, inhibits the production of glucagon, and stimulates the secretion of insulin in a glucose-dependent manner. Consequently, it may still be an appropriate agent for patients with relatively higher risk of hypoglycemia.

The potential safety issue of heart failure risk that arose from SAVOR-TIMI 53 and the Examination of Cardiovascular Outcomes with Alogliptin versus Standard of Care (EXAMINE) trials led to the US FDA’s recommendation [54] to consider discontinuing saxagliptin and alogliptin for patients if heart failure develops. However, according to our meta-analysis, risk of heart failure was similar with saxagliptin and comparators, with almost identical rates of heart failure in both groups. The result was opposite to previous systematic reviews and meta analyses, among which increased risk of heart failure was found in the treatment of saxagliptin individually or DPP-4 inhibitors as an integrity. Until now, there is no identified pathophysiology for the increased risk of heart failure by saxagliptin treatment. On the contrary, previous preclinical and mechanistic studies of DPP-4 inhibitors suggest additional nonglycemic beneficial actions on blood vessels and the heart, via both GLP-1-dependent and GLP-1-independent effects [55,56]. Positive effects of DPP-4 inhibitors on the myocardium have also been described in patients with ischemic heart disease [57].

US FDA had warned that DPP-4 inhibitors may cause serious arthralgia in August 2015, raising safety issues concerning the entire drug class and encouraging healthcare professionals and patients to pay attention [58]. In a search of the FDA Adverse Event Reporting System database, FDA has identified 33 cases of serious arthralgia reported with the use of DPP-4 inhibitors between October 2006 and December 2013. However, the current finding from our meta-analysis showed no safety signal for increased risk of arthralgia in the saxagliptin treatment, and a possible better effect than sitagliptin. Furthermore, there was no increased risk of pancreatitis and infections as supported by our studies, which was also consistent with previous meta-analyses [59–62]. Postmarketing safety surveillance of saxagliptin will provide further data on the incidences of adverse events.

In contrast to GLP-1 receptor agonists, saxagliptin does not mimic infused GLP-1 in its effects on subjective satiety or gastric volume, which have been associated with noticeable weight loss. Overall, saxagliptin appears to be associated with only modest changes in body weight. In comparison with placebo, saxagliptin was associated with a slight gain in body weight, which is of limited clinical significance. However, in comparison with sulfonylureas, saxagliptin had a significant advantage on body weight (−2.34 kg).

In the US and Europe, saxagliptin has been approved for use in patients with type 2 diabetes, both as monotherapy and in combination with metformin, a sulfonylurea, thiazolidinedione, or insulin, and also in combination with metformin plus insulin. However, in some countries like China, it is currently only approved for use as monotherapy or in combination with metformin. Our systematic review has demonstrated significant advantages of saxagliptin in achieving glycemic control when added to sulfonylureas, thiazolidinediones, or insulin, with similar or better safety profiles. These results will help administrators in China and other countries make evidence-based decisions.

This systematic review and meta-analysis of RCTs focused on the efficacy and safety of saxagliptin in the treatment of T2D. The sample size of nearly 30,000 patients enabled the demonstration of reliable results and the comparisons to most classes of oral antidiabetic drugs, by which the knowledge around the comparative efficacy and safety was enriched. However, there are some limitations in this study. Firstly, the follow-up periods of some included trials were relatively short, which limited the observation of longtime outcomes among some comparisons. Secondly, two trials included in the meta-analysis, which comparing saxagliptin with vildagliptin, sitagliptin and (or) liraglutide, were found to have possible high risk of performance and detection bias. This potential bias may reduce the credibility of corresponding results. Interpretations of these findings must be made with caution. More head-to-head trials between saxagliptin and active comparators are still needed to further confirm the efficacy and safety of saxagliptin compared with other classes of antidiabetic drugs.

In conclusion, Generally, saxagliptin has similar efficacy compared with most oral antidiabetic drugs, while may be more effective than acarbose. Saxagliptin is safe in the treatment of T2D, especially having a better safety profile than acarbose and sulfonylureas.

## Supporting information

S1 Text PRISMA checklist. (DOC)

S1 Fig Risk of bias of included trials (a. graph; b. summary). (EPS)

S2 Fig Patients achieved HbA1c<7% (a. saxagliptin vs placebo; b. saxagliptin vs active comparators). (EPS)

S3 Fig Serious adverse events. (EPS)

S4 Fig Body weight (a. saxagliptin vs placebo; b. saxagliptin vs active comparators). (EPS)

S5 Fig Other adverse events [a. pancreatitis; b. heart failure; c. arthralgia (saxagliptin vs placebo); d. arthralgia (saxagliptin vs active comparators); e. upper respiratory tract infection (saxagliptin vs placebo); f. upper respiratory tract infection (saxagliptin vs active comparators); g. urinary tract infection (saxagliptin vs placebo); h. urinary tract infection (saxagliptin vs active comparators); i. nasopharyngitis (saxagliptin vs placebo); j. nasopharyngitis (saxagliptin vs active comparators)]. (EPS)
